# Hospital Facts and Observations, &c.

**Published:** 1830-01-01

**Authors:** 


### XIII.

### Hospital Facts and Observations Illustrative of the Efficacy of the New Remedies Strychnia, Brucia, Acetate of Morphia, Veratria, Iodine, &c. in several Morbid Conditons of the System; with a Comparative View of the Treatment of Cholera, and some Cases of Diabetes, &c. By *James Lomax Bardsley*, M. D. Physician to the Manchester Infirmary, &c. &c. London, 8vo. pp. 224, 1829. Burgess and Hill, Great Windmill Street.

Dr. Bardsley appears to be a modest, intelligent, observant, and candid physician, who has had ample field for testing and comparing the effects of various remedies in various diseases, during his official duties at public institutions. In recording and publishing the results of his experience, he does the utmost in his power to further the interests of humanity, by increasing our knowledge or correcting our errors. Conscious of the mischief which has flowed, and which daily flows from the blazoning forth of successful cases, leaving the unsuccessful in the shade, Dr. Bardsley has made a point of detailing the unfavourable as well as the favourable cases treated more especially by that important class of substances the VEGETABLE ALKALIES, with the view of determining their real therapeutic properties. He has also made observations on the treatment of CHOREA and DIABETES by different remedies—diseases which appear to be very common at Manchester.

We shall glance at the several sections of the work, in which the author's experience is detailed.

I. *Strychnia in Paralysis.* It appears from the experiments of Desportes, Magendie, Delile, Orfila, and others, that the nux vomica does not occasion any organic lesion in the animal frame, though it has a direct action upon the nervous system, causing death from asphyxia produced by the immobility of the chest during the violence of the tetanic spasms of the thoracic and abdominal muscles. Fouquier was led to make trial of the strychnia in several cases of paralysis of the lower extremities, and published his successful results in that disease; and many others have employed the remedy with various, if not doubtful issues. Dr. B. details 23 cases of paralysis treated with strychnine, and gives a tabular view of twelve more. We shall glance at some of these in a condensed form.

*Case* 1. A female, aged 30, had entirely lost the power of the left side, with diminished sensibility, occasional head-ache and vertigo—articulation impaired—urine and fæces passed involuntarily—face drawn a little to one side—pulse feeble at 86—countenance pallid. The attack had occurred three months previously, shortly after parturition, and had gradually increased. Attributed the complaint to cold and over fatigue when far advanced in pregnancy. Leeches, blisters, and aperients having been premised, the strychnine was exhibited in doses of the twelfth part of a grain twice a day, and afterwards increased in frequency and quantity. It was more than three weeks before she began to feel the influence of the medicine, in slight pricklings of the paralyzed limbs. The sensation began to revive—smart convulsive twitchings occurred—motion improved—she had some command over the bladder and rectum—and finally recovered completely. The strychnine amounted to a grain twice a day, before the cure was effected.

"REMARKS. In this case the strychnia was very serviceable, and indeed, the patient's recovery was fairly attributable to a persevering use of this active remedy. The twelfth of a grain of the alkali was first exhibited twice a day, and this proportion was increased at regular intervals to the extent of one grain twice a day; but it was found that the patient could only take half a grain thrice in the day without experiencing a slight degree of inconvenience. The appetite was much improved during its exhibition." 10.

*Case* 2. This was a man aged 31 years, who, after being troubled with pain in the head for some months, lost the entire power of the lower extremities, their sensibility remaining unimpaired. The power over the sphincters was also lost—the flesh was reduced—the complaint of three months' duration—and the cause ascribed to fatigue and cold. Cupping and purging having been premised, the strychnine was exhibited, beginning with the fourth part of a grain three times a day, and gradually increasing the dose to half a grain three times a day, when convulsive twitchings were much complained of, but unaccompanied by vertigo or other distressing symptoms. The dose was ultimately augmented to double the above quantity, but severe pain at the scrobiculus cordis was the consequence, with violent spasmodic action of the muscles of the thorax and lower extremities, requiring powerful stimulants. Long and repeated trials were made of the medicine, but no benefit whatever was derived from its administration, and the patient was ultimately discharged uncured. The next case we shall quote in the words of the author.

"Case 3. Samuel Ogden, aged 46, admitted 21st June, 1824. He has been paralytic for more than four months, having lost the entire power of the limbs of the right half of the body; and the muscles of that side have not recovered their former bulk and plumpness. He has been addicted to great irregularities in his general mode of living. Complains of pain in front part of the head, accompanied by vertigo. Speech rather inarticulate. Memory slightly impaired. Urine and fæces are both involuntarily and unconsciously discharged. Appetite indifferent. Pulse feeble. Countenance somewhat sunk. Six leeches were ordered to each temple, and a blister to the nape of the neck. To take three ounces of the Mistura Sennæ Composita immediately, and to repeat the dose every three hours until the bowels have been freely evacuated. 23d. Head relieved by leeches and blister. Several copious stools procured by a second dose of the purging mixture. To commence with one-twelfth of a grain of strychnia in the form of a pill twice a day. 26th. Head continues free from pain. Bowels constipated. Paralytic parts remain in the same state. Dose of strychnia to be increased to the one-sixth of a grain three times a day, and the purging draught to be exhibited on alternate mornings. 29th. The only effect of the medicine as yet perceptible is an occasional sensation of heat along the spine. Bowels kept regular by draught. Dose of strychnia to be increased to the one-fourth part of a grain three times a day. 4th July. Slight convulsive twitchings of the paralyzed members. Head remains free from uneasiness. Thinks he possesses a little more feeling in the bladder and rectum. Dose of strychnia to be increased to half a grain twice in the day. Slight tetanic symptoms have been present after each dose of the medicine, but they are by no means alarming, or of long continuance. There appears to be some improvement in the affected limbs, for he can now raise both the arm and leg with a little assistance, which he was quite incapable of effecting on his admission. This circumstance affords him great satisfaction, and inspires him with hopes of recovery. Half grain pill to be taken three times a day. 7th. This quantity was more than the patient could bear, as it produced vertigo, stupor, pain at scrobiculus cordis, irregular convulsive startings both of the sound and paralyzed parts, tendency to syncope, weak pulse, and extreme debility. The pill was therefore ordered to be given only twice a day. He is now capable of gently moving both the affected arm and leg, and of retaining, during the day, his urine and fæces. To continue the pills. 10th. The diseased side improves. To persevere with the pills. 13th. The patient can now move both the arm and leg in several directions, and also support himself in the upright posture, by the help of a stick in the left hand. To have four ounces of wine daily and to continue the pills. 17th. Yesterday, with the assistance of crutches, he walked more than once across the ward, and he has acquired almost a full command over the muscles of the rectum and bladder. Pills to be continued. Wine to be increased to eight ounces in the day. The pills were regularly taken until the 1st of August, and the patient's amendment appears, from the last report up to this period, to have been progressive. On his discharge, he could walk from one end of the large ward to the other by the aid only of a small stick, and the functions of the bladder and rectum were completely restored.

"Remarks. This case affords evidence of the remedial efficacy of strychnia in palsy, and points out in a striking manner the *peculiar* action of the alkali upon the paralytic members, since no other remedies, with the exception of a few leeches to the temples, a blister to the nape of the neck, and occasional doses of aperient medicine (on the patient's first admission) were employed. I had my doubts respecting the propriety of administering the alkali in this instance, as there was some reason to fear, from the previous intemperate habits of Ogden, that the hemiplegia was owing to disorganization of the brain; hence I adopted the precaution of relieving the vessels of the head before I commenced with the use of the strychnia. One grain during the day was as much as he could take with safety, for when the dose was increased to a grain and a half daily, very unpleasant effects followed. It would have been improper to have pushed the remedy beyond the limits pointed out by the symptoms. It may be well to notice the *sensation of heat along the course of the spine* more than once experienced by the patient, and first mentioned by himself, without the question having ever been put to him, as it tends to confirm the opinion before stated, respecting the peculiar action of the strychnia upon the spinal chord." 20.

*Case* 4. This was a spinner, aged 29 years, who, six months previously was seized with loss of power in the lower extremities after bathing whilst the body was much heated with exercise. At the time of admission he was incapable of motion without the aid of crutches, and passed his urine and fæces involuntarily. The spine was free from pain—strength much reduced—appetite bad. The strychnine, in the dose of one-sixth of a grain three times a day, was exhibited, and augmented to a quarter of a grain every four hours, when severe convulsive twitchings took place in the affected limbs. In less than a month he became capable of retaining both his urine and fæces, and of walking from one end of the ward to the other by the aid of a small stick. He was entirely cured.

*Case* 5. Sarah Hilton, aged 36 years, was admitted on the 7th September. This woman had suddenly lost the power of the right side of the body. The attack was preceded by severe pain at the crown of the head, drowsiness, faulty articulation, &c. The intellectual faculties were impaired, as was the power over the sphincters. She was ordered five grains of calomel, followed by a saline aperient, which were repeated, and then the strychnine was exhibited, in the dose of a quarter of a grain every six hours. This was gradually increased till violent twitchings of the paralytic parts ensued, attended by nausea and dyspnœa. These symptoms were relieved by plenty of brandy and water. The medicine was soon afterwards resumed and continued. The power of motion in the paralyzed parts soon began to return—the amendment was rapid—and in about seven weeks the cure was complete.

Passing over a case or two, we shall extract the following, without abbreviation.

"Case 6. Robert Hobson, 38 years of age, finisher, admitted an in patient, October 6, 1827.

"This is a case of paraplegia, the patient having lost the power over the inferior extremities, rectum, and bladder. He states that he first perceived about four months ago a weakness in his legs, rendering it necessary for him to use considerable effort to drag them along. This debility gradually increased, until at length he became altogether incapable of moving the lower limbs. He has no feeling in them. Head free from pain or giddiness. Pulse regular, appetite impaired. Being desirous of putting the *individual* efficacy of the strychnia to the test, I commenced with the exhibition of the alkali in the proportion of a sixth of a grain twice daily, without previously employing internal or local remedies of any kind. October 10th. No change. Dose to be increased to the fourth of a grain three times in the day. 20th. He considers himself better, having obtained a slight command over the sphincters of the bladder and rectum. On the 14th, he first experienced involuntary twitchings in the inferior extremities, which have been continued at intervals up to the present time. To take half a grain of the alkali twice daily. 28th. He is in excellent spirits, owing to the benefit he has derived from the pills. He can raise the lower limbs to some height from the bed, and also retain at pleasure both his urine and fæces. During the last week, the twitchings have been rather severe, but not painfully so. He is very desirous of persevering with the pills. To continue. November 7th. Since the last report, the patient has been allowed to sit up for several hours during the day. He is surprisingly improved, being able to walk without the aid of a stick from one end of the long ward to the other. Appetite good. Bowels regular. Warmth and sensibility of inferior extremities natural. Half grain pill to be taken three times daily. 26th. The additional half grain excited for some days powerful twitching in the legs and thighs, but in the course of a week or less they became not more severe than was occasioned by half a grain of the alkali taken twice in the day. He is now capable of walking as well as at any former period of his life, and his general health is excellent. It is impossible to describe the gratitude which this patient felt for his restoration to health. He was ordered to be discharged *cured* at the first meeting of the weekly board." 29.

Our author next states the effects of the strychnia in thirteen cases of paralysis; but, in order to avoid tiresome details of history, he merely mentions the age of the patient, duration of the disease, time of admission, dose of alkali employed, length of time it was continued, and the result. This abbreviation we shall here introduce, as it harmonizes with our own plan of brevity.

"Case XI. August 4th, 1825, Elizabeth Taylor, aged 38.

"Has been paralytic on the right side for two months. Attack sudden, accompanied with pain in the head. To commence with the eighth of a grain of strychnia twice daily. Gradually increased to half a grain twice in the day. Continued this dose for three months. Discharged cured.

"Case XII. September 2d, 1825, Sarah Whyatt, aged 53.

"Ill six months. Hemiplegia of right side. Fourth of a grain of strychnia to be taken twice daily. Increased to half a grain, at the same intervals. Continued for four months. Discharged relieved.

"Case XIII. October 4th, 1825, William Johnson, aged 16.

"Paraplegia of six weeks standing. Cause of disease unknown. Sixth of a grain of alkali to be taken twice in the day. Increased to the fourth of a grain. Persevered in its use for two months. Discharged cured.

"Case XIV. December 30th, 1825, Thomas Griffiths, aged 61.

"About two months ago was seized with hemiplegia of the left side. Former habits intemperate. Head painful. Ten leeches to be applied to temples. Pain in the head removed by leeches. Sixth of a grain of strychnia to be given twice in the day. Increased to half a grain. Continued three months. Discharged relieved.

" CASE XV. January 4th, 1826, ANN LLOYD, aged 42.

" Has been paralytic on the left side for more than six months. Commenced with the eighth of a grain of alkali. Increased to half a grain three times daily. Continued four months. Discharged cured.

" CASE XVI. January 15th, 1826, THOMAS KNOTT, aged 50.

" Hemiplegia of left side. Experienced the attack about twelve months ago. Sixth of a grain of strychnia to be taken each morning and evening. Increased to a grain and a half during the day, Persevered in the use of the remedy with great regularity for three months. Discharged much relieved.

" CASE XVII. April 2d, 1826, THOMAS OGDEN, aged 40.

" Has laboured under paralysis of right side for two months. To commence with the sixth of a grain of alkali twice in the day. Increased to half a grain. Continued two months. Discharged cured.

" CASE XVIII. September 3d, 1826, JOHN LEVERS, aged 46.

" Paraplegia of three months' continuance, induced by a severe fall upon the sacrum. Sixth of a grain of strychnia twice daily. Increased to half a grain. Remained in the hospital two months. Discharged cured.

" CASE XIX. August 19th, 1826, MARY GRADY, aged 62.

" Lost the power of right side four months ago. Fourth of a grain of alkali twice daily. Increased to half a grain. Three months trial of the medicine. Discharged relieved.

" CASE XX. October 6th, 1826, WILLIAM SICKSMITH, aged 39.

" Paralytic on right side five months. Fourth of a grain of strychnia twice daily. Increased to a grain and a half in the course of a day. Discharged cured in less than ten weeks from the time of admission.*

" CASE XXI. December 28th, 1826, ——— JONES, aged 33.

" Lost power of right arm about a year and a half ago. Eighth of a grain of strychnia three times in the day. Gradually increased to half a grain twice daily. Two months trial of alkali. Discharged cured.

" CASE XXII. February 4th, 1827, JAMES WILSON, aged 29.

" Attacked suddenly in March last with paraplegia after exposure to wet and cold. Dose of strychnia to be increased from sixth of a grain to half a grain twice daily. Used this remedy for one month. Discharged cured.

" CASE XXIII. November 4th, 1827, JAMES TURNER, 48 years of age.

" Has laboured under hemiplegia of left side for five weeks. Commenced with the sixth of a grain twice daily. Dose increased to half a grain. Remained two months in the hospital. Discharged cured.

" The following table will also shew the results of the exhibition of strychnia in *twelve* more cases of paralysis.

---

* " This man has since experienced an attack of paraplegia, and is now using the strychnia (at his own request) with advantage. March 10th, 1828."

| No. | Names. | Age. | Duration of complaint. | Disease. | Result. |
|---|---|---|---|---|---|
| 24 | James Faris, I. P.* | 29 | 3 months. | Hemiplegia of left side . | Cured. |
| 25 | Charles Walker, I. P. | 44 | 6 months. | Ditto. | Much relieved. |
| 26 | Robert Ashton, H. P. | 54 | 2 years. | Paraplegia . | Slightly relieved. |
| 27 | Margaret Lewis, O. P. | 17 | 2 months. | Ditto. | Cured. |
| 28 | Denis Hagan, H. P. | 44 | 6 weeks. | Hemiplegia of right side | Much relieved. |
| 29 | Henry Black, I. P. | 23 | 3 weeks. | Paraplegia from a fall . | Cured. |
| 30 | John Allensworth, O. P. | 62 | 12 months. | Hemiplegia of right side | No benefit. |
| 31 | Isabella Gale, I. P. | 33 | 3 months. | Ditto. | Left the house. |
| 32 | David Davis, H. P. | 51 | 3 months. | Hemiplegia of left side . | Much relieved. |
| 33 | Jane Shaw, H. P. | 19 | 4 weeks. | Paraplegia from cold . . | Cured. |
| 34 | Owen Morris, O. P. | 54 | 7 months. | Hemiplegia of left side . | Relieved. |
| 35 | Jacob Long, O. P. | 49 | 12 months. | Paraplegia . | Cured. |

### Observations.

Our author thinks, and we agree with him, that the foregoing recital is sufficient to prove that strychnia excites a very powerful influence on the system, and that it is entitled to rank as a valuable remedy in the materia medica. It was employed in some cases without benefit, in others with partial advantage—but, in the majority, with complete success. Hence, he conceives, it may justly be considered an *efficacious*, though not a certain remedy in this affection. When cerebral disorganization has once taken place, it is, of course, in vain to expect a cure from any medicine. "It is in such cases of paralysis as seem to arise from diminished nervous excitement, that the strychnia is particularly indicated."

"It may be stated here, as a rule of guidance, that whenever hemiplegia supervenes to an apoplectic seizure in persons of a plethoric habit, it is proper to employ bleeding, purging and the ordinary antiphlogistic treatment, before resorting to the use of the strychnia. When the vessels of the head have been freely unloaded, and the quantity and force of the circulating fluid diminished by the above means, there can be little objection to a *cautious* and *prudent* trial of this remedy. Generally speaking, the strychnia is likely to prove more serviceable in paraplegia, unconnected with spinal disease, than in hemiplegia; though I feel confident, that it will not unfrequently be found an important remedial agent even in hemiplegiac paralysis." 39.

* "The abbreviations, I.P. H.P. and O.P. denote in-patients, home-patients, and out-patients."

Dr. B. has not tried the strychnine on children, nor would he advise the attempt. The first effects of the medicine, in every case, were convulsive twitchings of the paralytic members, and no benefit was derived until this condition of the parts had been produced and continued for some time. This appears to have been the case with iodine in the hands of that very intelligent and candid physician, Dr. Manson. In Dr. Alderson's experiments, too, with the rhus toxicodendron in paralysis, twitchings and tinglings in the paralytic members appear to have been the first steps towards recovery. Strychnia does not appear to impair the energy of the stomach, but is rather serviceable in promoting appetite and digestion. Dr. B. recommends the practitioner to commence with not more than the eighth of a grain of strychnine twice in the 24 hours, which quantity is to be gradually increased to a sixth, fourth, or even half a grain at the same intervals. Any unpleasant symptoms should be the signal for suspending the remedy for a time, when it is to be renewed in slowly augmented doses. "By attention to these points (says our author) although no benefit may accrue from the strychnine, we may be sure that no injury will attend its exhibition." He generally gave it in a pill, with a little conserve of roses.

II. *Strychnia in Chronic Diarrhœa.* Numerous instances of chronic diarrhœa occur among the out-patients of the Manchester Infirmary, some of which obstinately resist every kind of treatment. Under such circumstances, our author was induced to try the strychnia, "and it proved a *safe* and *effectual* remedy." To shew the foundation for this assertion Dr. B. has detailed several cases in illustration. Since he had prepared these pages for the press, he finds that Recamier had also exhibited the alcoholic extract of nux vomica with complete success. Dr. Rummel has also employed strychnia with advantage in chronic blennorrhœa of the rectum. A single case will suffice as an example.

"Case III. John Neary, 55 years of age, admitted an out-patient, March 16th, 1826.

"He had been formerly addicted to the intemperate use of spirituous liquors, but of late years his habits have been regular and sober. He complained of a frequent desire to go to stool, having not less than five or six evacuations daily, and his strength had declined during the last month very rapidly. His appetite was impaired, and he had copious perspirations in the night. He attributed his complaint to exposure to wet and cold in January last. He had tried several remedies, but without benefit. The ordinary astringents were unsuccessfully administered. I then commenced with the strychnia, which succeeded, as the reports will shew, in speedily restoring the patient's health. He began on the 18th, with the sixth of a grain of the alkali, night and morning, which was increased to the fourth of a grain three times a day. On the 10th of April he had not more than two stools daily. His appetite was better, and he had gained strength. He continued this plan of treatment until the 24th, when he was discharged cured. I saw this man about a year afterwards, and he informed me that he had not suffered any relapse of his disorder." 46.

In his remarks on the cases detailed, Dr. B. observes that he does not consider the strychnia a suitable remedy in those instances of diarrhœa which depend upon an evident inflammation of the mucous membrane of the intestines. He particularly recommends the medicine in cases of a *chronic kind*, occurring in persons somewhat advanced in life, and of feeble constitution.

III. *Strychnia in Amenorrhœa.* This complaint is the source of many others of a very troublesome character, and is, moreover, one of very difficult management. It is therefore of importance to be able to name any new remedy which may be found occasionally serviceable in promoting a regular and natural flow of the catamenia.

" Previously to my experiments with the strychnia in this disease, I had been in the habit of employing aloes more extensively than any other medicine ; and I can add my testimony to that of the venerable Dr. Hamilton, in favour of the utility of purgatives in exciting the action of the uterine vessels. In many cases, I have succeeded in obtaining a renewal of the menstrual discharge, by a steady but active administration of the aloetic pill *alone*." 51.

We shall glance at some of the cases.

*Case* 1. Phœbe Love, 33 years of age, unmarried, June 4th. She stated that she had not menstruated for five months past, the obstruction being attributed to cold. She appeared languid and weak—appetite impaired—countenance pallid—bowels costive. These last being brought into a proper state by aperients, the sixth of a grain of strychnine was administered three times a day. On the 16th June the dose was increased, there being no perceptible effect produced. The report of the 12th July states that the general health is much improved—that she had felt some painful sensations in the hypogastric region during the last few days. On the 24th the menses appeared. The discharge continued regular afterwards, and she is now the mother of two children. We are unable to state any more of the cases, but shall present the following tabular view of eight cases not contained among the detailed accounts.

| No. | Name. | Age. | Duration of Complaints. | Result. |
|---|---|---|---|---|
| 1 | Mary Holfroys ..... .. | 44 | 9 months ........ | Cured. |
| 2 | Margared Cain .......... | 29 | 5 months ........ | Ditto. |
| 3 | Sarah Hooley......... ... | 24 | 12 months ........ | Ditto. |
| 4 | Jane Marsden . ........ | 40 | 4 months .... | Ditto. |
| 5 | Mary Ferguson .......... | 19 | 6 months ..... .. | Ditto. |
| 6 | Elizabeth M'Avoy........ | 35 | Ditto . . .. .. . | Relieved. |
| 7 | Jane Wragg ........... | 38 | 18 months ..... . | Cured. |
| 8 | Elizabeth Corr ..... ... | 29 | 2 years.......... | Relieved. |

The cases appear to our author sufficient to shew the remedial virtue of strychnine in some instances of amenorrhœa, owing, he thinks, to the power which the alkali possesses of stimulating the vessels of the uterus, and improving the tone and vigour of the system. Dr. B. advises the conjoint exhibition of mild laxatives with the alkali in this affection, when the bowels, as is most commonly the case, are constipated.

" Such are the results of my experiments with strychnia, which are calculated to set forth the *real* claims of this alkali to the notice of the profession, as a remedy in certain diseased conditions of the system. I think that I may venture to draw from them the two following conclusions. First, that strychnia, *cautiously administered,* is a safe and useful remedy in paralysis. Secondly, that it will *occasionally* be found serviceable in *chronic diarrhœa* and *amenorrhœa.*" 58.

Dr. B. has appended several cases of paralysis treated by brucia, an alkali obtained also from the nux vomica, and weaker than the strychnine in the proportion of one to twelve. Ten cases are selected from among others, to show the powers of the brucia. His experience leads him to recommend it as a valuable remedy in paralysis—the action being analogous to that of strychnine, but less powerful—" hence," says he, " it is a preferable remedy in paralytic attacks, accompanied with much cerebral disturbance." It is prudent to commence with the dose of a grain twice a day, which is to be cautiously increased to two grains three or four times a day. Unless a marked advantage accrue from the administration of the remedy in the course of five or six weeks, it ought to be laid aside.

This brings us to about the middle of the volume, and as the remaining subjects of discussion are totally unconnected with that which we have now concluded, we shall defer our analysis of those topics till our next number. By this procedure we do not break the thread of any narrative, nor the unity of any investigation entered into by the intelligent author of the work under review.

